# Calcific constrictive pericarditis following tumour necrosis factor-alpha inhibition

**DOI:** 10.1093/ehjcr/ytae027

**Published:** 2024-01-20

**Authors:** Brian Cunneen, Hafiz Hussein, Jenna O’Sullivan, Ibrahim Yearoo

**Affiliations:** Cardiology Department, Beaumont Hospital, Beaumont Road, Dublin 9, Republic of Ireland; Cardiology Department, Beaumont Hospital, Beaumont Road, Dublin 9, Republic of Ireland; Cardiology Department, Beaumont Hospital, Beaumont Road, Dublin 9, Republic of Ireland; Cardiology Department, Beaumont Hospital, Beaumont Road, Dublin 9, Republic of Ireland

**Keywords:** Case report, Constrictive Pericarditis, Adalimumab, TNF-alpha inhibition, Pericardectomy

## Abstract

**Background:**

Tumour necrosis factor (TNF)-alpha inhibition is a core therapeutic avenue for a broad range of inflammatory and autoimmune disorders including rheumatoid arthritis and inflammatory bowel disease, as well as dermatological conditions such as hidradenitis suppurativa. Adalimumab has become one of the most common TNF-alpha–inhibiting agents, which is used for many of these conditions. Treatment with such agents is associated with numerous systemic side effects, though cardiac complications remain relatively rare. These include reports of pericarditis and pericardial effusions^1–3^.

**Case summary:**

A 63-year-old lady was referred to the outpatient respiratory clinic with a 1-year history of increasing breathlessness, on a background of 4 years of treatment with adalimumab for Stage III hidradenitis suppurativa. A high-resolution computed tomography (CT) thorax revealed evidence of pericardial calcification. Subsequent left and right heart catheterization study revealed equalization of intraventricular pressures, consistent with constrictive pericarditis. A QuantiFERON test was negative, and rheumatological serology was unremarkable. The patient was initially managed conservatively with close follow-up, before undergoing surgical pericardectomy when she developed signs of cardiac failure.

**Discussion:**

Adalimumab is associated with a range of systemic side effects, though cardiac complications are relatively rare. This case highlights a potentially novel complication associated with prolonged adalimumab therapy. Given that there are reports in the literature of pericarditis and pericardial effusions associated with TNF-alpha inhibition^1–3^, it is reasonable to hypothesize that the calcific constrictive pericarditis seen in this case may demonstrate a novel cardiac phenomenon associated with this therapy, given the lack of any traditional aetiological factors.

**Learning points**

- To develop an understanding of the presentation, aetiology, and management of constrictive pericarditis.
- To be aware that N-terminal pro B-type natriuretic peptide can be disproportionately normal in heart failure patients with constrictive pericarditis.
- To identify factors leading to poor prognostic outcomes in constrictive pericarditis.

## Introduction

Tumour necrosis factor (TNF)-alpha inhibition is a core therapeutic avenue for a broad range of inflammatory and autoimmune disorders including rheumatoid arthritis and inflammatory bowel disease, as well as dermatological conditions such as hidradenitis suppurativa. Adalimumab has become one of the most common TNF-alpha–inhibiting agents, which is used for many of these conditions. Treatment with such agents is associated with numerous systemic side effects, though cardiac complications remain relatively rare.^1–3^ We present the case of a 63-year-old woman with calcific constrictive pericarditis (CP) following prolonged adalimumab therapy, with no other identifiable cause.

## Summary figure

**Table:** 

Date	Event
June 2017 to September 2021	Receiving adalimumab for hidradenitis suppurativa
November 2021	Reviewed in the outpatient department with New York Heart Association II dyspnoea. Subsequent chest x-ray demonstrates pericardial calcification
July 2022	Undergoes right and left heart catheterization, revealing constrictive physiology. Patient is expectantly managed, given low symptom burden
November 2022	Patient reports progressive breathlessness. Computed tomography thorax performed, which is indicative of decompensated heart failure
March 2023	Undergoes pericardiectomy. Clinically well post-operatively

## Case presentation

A 63-year-old lady was referred to the outpatient respiratory clinic with a 1-year history of increasing breathlessness, on a background of recent adalimumab therapy for Stage III hidradenitis suppurativa (HS). She had been on adalimumab for 4 years in total before it was ceased, for satisfactory remission of her HS. At the time of presentation, she had been off adalimumab for 4 months.

**Figure 1:**
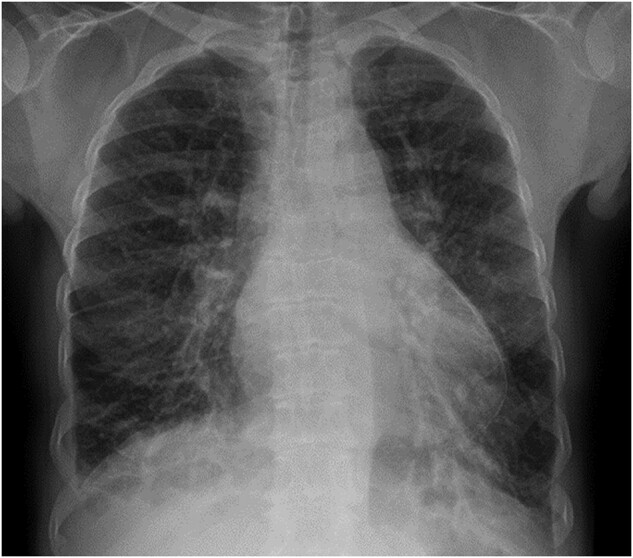
Initial chest x-ray demonstrating pericardial calcification.

**Figure 2:**
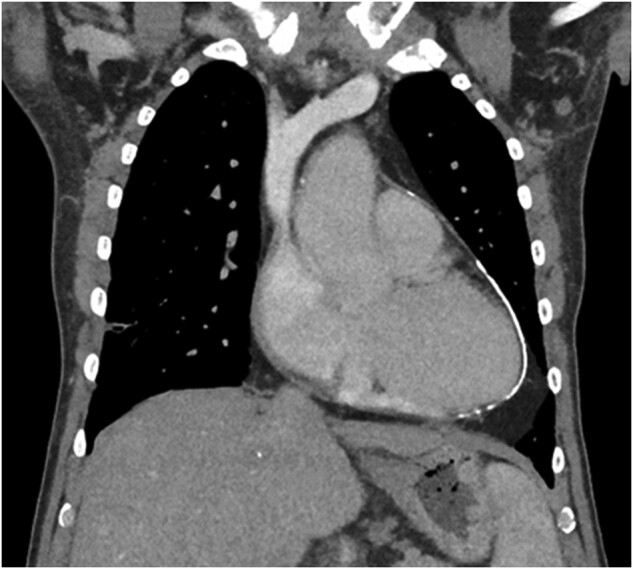
Coronal view of computed tomography thorax, demonstrating pericardial calcification.

**Figure 3:**
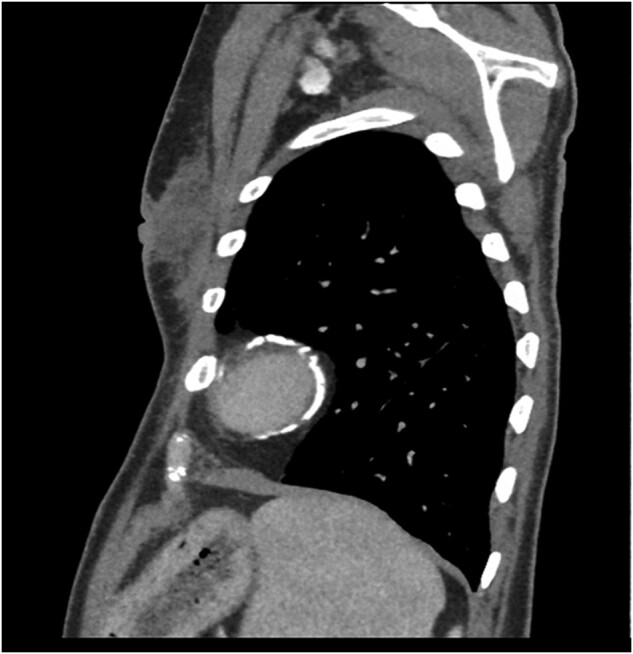
Sagittal view of computed tomography thorax demonstrating circumferential calcification of the apical region of left ventricle.

**Figure 4:**
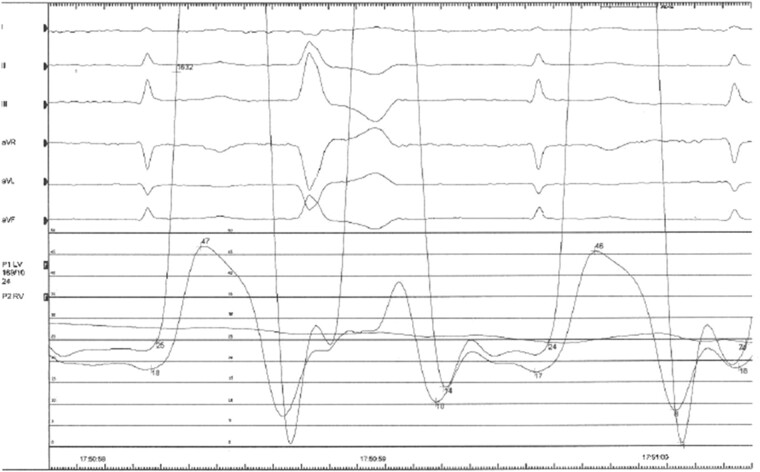
Invasive haemodynamic tracings, demonstrating equalization of intraventricular end-diastolic pressures.

**Figure 5:**
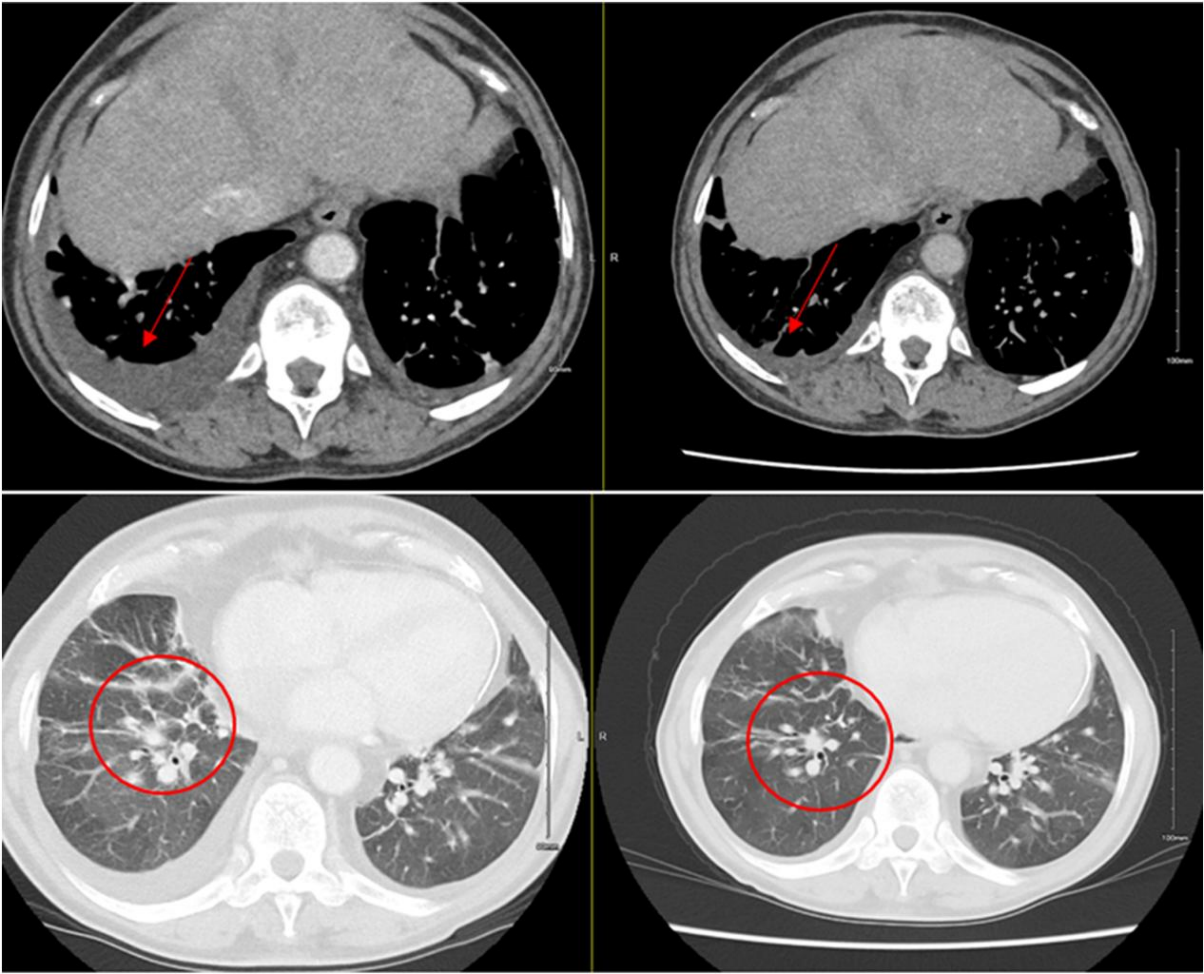
Comparison of pleural effusions and peri-bronchial thickening between initial and subsequent computed tomography thorax, indicating worsening heart failure.

On assessment, she had New York Heart Association III dyspnoea. She had not suffered from any orthopnoea or recent chest pain. Clinical examination from a respiratory, cardiac, and rheumatological perspective was unremarkable, and her vital signs were within normal limits.

The patient had a background history significant for Hurley Stage III HS for which she had been on adalimumab for 4 years. She was an ex-smoker of 18 years with approximately 20 pack years of history. She had no other past respiratory or cardiac history.

The patient had a chest x-ray and subsequent high resolution computed tomography (CT) thorax performed. These investigations demonstrated significant pericardial calcification, primarily over the left ventricle (LV), with the posterior and lateral walls particularly affected. The calcification continued to become circumferential at the apex (*Figures 1–3*).

A transthoracic echocardiogram revealed Grade I diastolic dysfunction but was otherwise unremarkable. Aetiological workup of these findings included a QuantiFERON, as well as a rheumatological panel incorporating an autoantibody and myositis screen, all of which were negative. She had no clinical features of connective tissue disease and no prior history of cardiac surgery, pericardial disease, or radiation therapy.

She then underwent invasive right and left heart catheterization studies, which demonstrated equalization of right-sided and left-sided end-diastolic pressures, consistent with a diagnosis of CP (*Figure 4*).

Following multi-disciplinary discussion, conservative management was recommended with close monitoring, given that her symptoms had begun to improve since initial presentation. The considerable morbidity and mortality associated with surgical intervention was, in discussion with the patient, weighed up against her relatively mild symptom burden, and it was agreed to manage her case expectantly.

The patient was being closely followed on a 3-month basis to monitor for any symptom progression, which would likely represent an indication to proceed with pericardiectomy. Unfortunately, 9 months after her diagnosis, she once again developed increasing breathlessness.

A repeat CT thorax revealed worsening bibasilar peri-bronchial opacities and probable inter-lobular septal thickening, likely related to chronic cardiac congestion with atelectasis. There was also radiological evidence of liver cirrhosis, possibly secondary to congestive hepatopathy. In addition, this CT also demonstrated extensive pericardial calcification with dilation of the hepatic veins, suggestive of chronically elevated right heart pressures. Furthermore, a moderate right pleural effusion and a new small left pleural effusion had developed (*Figure 5*).

The above radiological findings correlated with clinical examination, confirmed evidence of right heart failure. Of note, all her biochemical markers remained normal, including an N-terminal pro B-type natriuretic peptide (NT-proBNP) of 182 pg/mL.

She was re-discussed at our Heart Team meeting, and early pericardiectomy was advised while her biventricular function remained normal. She was prescribed a low-dose loop diuretic under close supervision and subsequently underwent surgical pericardiectomy. She recovered well in the post-operative period. Of note, despite significant improvement in her symptoms, her NT-proBNP rose post-operatively to 904 pg/mL.

## Discussion

Calcific CP is an uncommon condition in which calcification of the pericardium restricts the normal diastolic filling within the heart. Its aetiology is usually associated with prior tuberculosis, trauma from cardiac surgery, or radiation therapy. It can occasionally occur in patients with recurrent episodes of pericarditis.

The diagnosis of calcific CP is made using a combination of radiological imaging to demonstrate calcific morphology of the pericardium, as well as functional testing to identify haemodynamic changes.^4^

It has been reported that patients with CP have disproportionately normal or mildly elevated BNP values compared with their clinical symptoms and signs of heart failure. This has been attributed to lesser stretching of the cardiac myocytes due to the constrictive effect of the diseased pericardium.^5^ It is worth noting that in this case, the NT-proBNP remained normal until the pericardectomy had been performed.

The only definitive treatment for CP remains surgical pericardiectomy. This is recommended early in the disease course, before the patient develops severe symptoms, as this is associated with improved outcomes.^6^ Survival is dependent on the underlying aetiology, and patients with idiopathic CP tend to have better outcomes after pericardiectomy.^7^

Pericardiectomy is however associated with a 5–10% peri-operative mortality.^8^ The surgical risk in patients with advanced diseases or those with LV systolic dysfunction is considerably higher, with an estimated 40–60% peri-operative mortality rate.^9^

Pericardial complications arising with TNF-alpha inhibition, while uncommon, have been reported in the literature. These include pericardial effusions and pleuropericarditis.^1–3^ As such, while calcific CP has not previously been linked with adalimumab, it is reasonable to suggest it may also predispose patients to this condition. The patient described in this case had a history of prolonged adalimumab therapy and no identifiable cause of CP. This would represent a novel association between this medication and a cardiac condition, which can be particularly challenging to manage. At the time of writing, numerous biosimilars to adalimumab are currently in development. While cardiac complications associated with this medication are rare at present, these may become more prevalent in the coming years with ever increasing use of such therapy.

## Conclusions

TNF-alpha inhibition with adalimumab has become one of the core treatment avenues for a range of inflammatory and autoimmune disorders. Though cardiac complications remain relatively uncommon, there exists reports in the literature of conditions such as pericardial effusions and pleuropericarditis, linking them to these medications.^1–3^ This case demonstrates the occurrence of calcific CP in a patient without any of the more typical risk factors such as previous tuberculosis, cardiac surgery, or thoracic irradiation. Given the reports of pericardial complications associated with adalimumab in the literature, this may represent a potentially novel aetiology of this patient’s presentation.

## Data Availability

The data underlying this article is available in the article and in its Supplementary material online.

## Lead author biography

I am a Cardiology Specialist Registrar from Ireland. I have always been passionate about the role of cardiovascular imaging in healthcare. I intend to pursue a career in heart failure and cardiovascular imaging.

**Consent:** The authors confirm that the written consent for submission and publication of this case report, including images and associated text, has been obtained from the patient in line with COPE guidance.

**Funding:** None declared.
